# Unveiling the Subtle Threats: The Neurobehavioral Impact of Chlorpyrifos on *Girardia tigrina*

**DOI:** 10.3390/toxics12070512

**Published:** 2024-07-15

**Authors:** Althiéris S. Saraiva, Eloisa Borges dos Reis, Fernanda S. Farnese, Marilene S. Oliveira, Pearl U. Ofoegbu, Aline S. P. Dornelas, Renato A. Sarmento, João C. P. de Souza, Erika C. Resende, Andreia C. M. Rodrigues

**Affiliations:** 1 Instituto Federal de Educação, Ciência e Tecnologia Goiano—Campus Campos Belos (CAE Research Group—Conservação de Agroecossistemas e Ecotoxicologia), Campos Belos 73840-000, GO, Brazil; 2 Instituto Federal de Educação, Ciência e Tecnologia Goiano—Campus Rio Verde, Rio Verde 75901-970, GO, Brazil; eloisaborgesreis@gmail.com (E.B.d.R.); fernanda.farnese@ifgoiano.edu.br (F.S.F.); marilenes36@gmail.com (M.S.O.); 3 CESAM—Centre for Environmental and Marine Studies, Department of Biology, University of Aveiro, 3810-193 Aveiro, Portugal; pearl.ofoegbu@ua.pt; 4 Programa de Apoio à Fixação de Jovens Doutores no Brasil, Estagio Pós-Doutoral—Universidade Federal do Tocantins, Campus Universitário de Gurupi, Gurupi 77402-970, TO, Brazil; eng.alinesilvestre@gmail.com; 5 Programa de Pós-Graduação em Produção Vegetal, Universidade Federal do Tocantins, Campus Universitário de Gurupi, Gurupi 77402-970, TO, Brazil; rsarmento@mail.uft.edu.br; 6 Departamento de Química da Faculdade de Ciências da Universidade Estadual Paulista “Júlio de Mesquita Filho”—Unesp—Campus Bauru, Bauru 17033-360, SP, Brazil; joao.perbone@unesp.br; 7 Instituto Federal de Educação, Ciência e Tecnologia Goiano—Campus Iporá-GO, Iporá 76200-000, GO, Brazil; erika.resende@ifgoiano.edu.br

**Keywords:** ecotoxicity, organophosphate insecticide, freshwater planarians

## Abstract

Chlorpyrifos, an organophosphate insecticide widely used to control agricultural pests, poses a significant environmental threat due to its toxicity and persistence in soil and water. Our work aimed to evaluate the acute (survival) and chronic (regeneration, locomotion, and reproduction) toxicity of chlorpyrifos to the non-target freshwater planarian *Girardia tigrina*. The 48 h lethal concentration (LC_50_) of the commercial formulation, containing 480 g L^−1^ of chlorpyrifos, the active ingredient, was determined to be 622.8 µg a.i. L^−1^ for planarians. Sublethal effects were translated into a significant reduction in locomotion and delayed head regeneration (lowest observed effect concentration—LOEC = 3.88 µg a.i. L^−1^). Additionally, chlorpyrifos exposure did not affect planarian fecundity or fertility. Overall, this study demonstrates the potential of chlorpyrifos-based insecticides to harm natural populations of freshwater planarians at environmentally relevant concentrations. The observed toxicity emphasizes the need for stricter regulations and careful management of chlorpyrifos usage to mitigate its deleterious effects on aquatic ecosystems. By understanding the specific impacts on non-target organisms like *G. tigrina*, we can make more informed suggestions regarding the usage and regulation of organophosphate insecticides, ultimately promoting sustainable agricultural practices and environmental conservation.

## 1. Introduction

Areas of intensive agricultural production have been reported to contribute considerably to the contamination of aquatic ecosystems, mainly due to the intensive use of pesticides [1]. Generally, intensive agriculture areas occur mainly close to water sources, and several studies have documented the occurrence and quantification of pesticides in surface waters adjacent to areas of high agricultural activity [2,3,4,5].

Among the vast diversity of pesticides commonly used in agriculture is chlorpyrifos (O,O-diethyl O-3,5,6-trichloropyridin-2-pyridinyl phosphorothioate), an organophosphate insecticide and biocide. Chlorpyrifos is used to control termites, pests affecting a variety of agricultural crops such as rice, fruits, vegetables, tobacco, cereals, nuts, mushrooms, cotton, and ornamental plants, pests of animals, and other pests found in and around residential areas [6,7]. It is widely used in agriculture to control insect pests of families Aphididae, Eriophyidae, Noctuidae, and Pyralidae [8]. Chlorpyrifos acts on the nervous system of insects, causing nervous disorder and incoordination through inhibition of the activity of acetylcholinesterase (AChE), overaccumulation of acetylcholine, and stimulation of postsynaptic muscarinic and nicotinic receptors [9].

Chlorpyrifos has been reported to reach surface water mainly by runoff, erosion, tail waters, irrigation water, and aerial spray, and can leach into groundwater [10,11,12]. It dissipates easily in the environment with moderate persistence in surface water, with the capacity to be carried over long distances, is persistent in water sediments, has low solubility in water, is biodegradable (microbial degradation), can undergo chemical hydrolysis, clay-catalyzed hydrolysis, and volatilization, and has a half-life of up to 140 days under neutral conditions [7,13]. Environmental concentrations of about 37.3 μg L^−1^ in surface water, 2.00 µg L^−1^ in groundwater, and 12 µg Kg^−1^ in water bottom sediment a day after application in a farm, effluents, and sludge have been reported [13,14,15,16].

The U.S. Environmental Protection Agency (EPA) revised in 2016 the chlorpyrifos human health risk assessment using neurodevelopmental effects as the critical endpoint for risk assessment, indicating increased concern about these impacts [17]. Recent studies have corroborated ongoing exposure risks in some regions, particularly for children and specific populations [18]. Recognizing the ecological and human health risks associated with chlorpyrifos, restrictions on its use have been implemented in Europe, the United Kingdom, and the United States of America, among others. However, it continues to be utilized in other countries, including Brazil, Russia, India, and China [14,19,20,21]. A study utilizing a species sensitivity distribution analysis reported acute toxicity of chlorpyriphos in 207 species of aquatic organisms, including vertebrates and invertebrates [22]. The potential toxic impacts caused by exposure to chlorpyriphos include unbalanced oxidative stress, morphological and behavioural changes, alterations of enzyme activity, neurotoxicity, endocrine dysfunction, and apoptosis [20,23]. To enhance our understanding of the ecological risks posed by chlorpyrifos and to develop suitable risk management strategies, international agencies like the European Chemicals Agency (ECHA) are requesting additional ecotoxicological data to refine their advisory recommendations [12].

The planarian species *Girardia tigrina* (Girard, 1850) (Paludícola: Dugesiidae) has been recently utilized in ecotoxicological studies and possesses significant potential as a bioindicator for environmental contamination, owing to its distinctive physiological and behavioral characteristics [24,25,26,27,28]. Planarians exhibit desirable traits for testing purposes, as they serve both as predators and prey, and can be easily monitored in laboratory settings [27]. Furthermore, planarians have gained prominence in international research across various scientific fields, including pharmacology and ecotoxicology, due to the ease and efficiency of conducting experiments with these organisms when compared to other alternatives and due to the similarities between their nervous systems with those of mammalians [29,30,31]. Additionally, planarians are widely employed in scientific investigations focused on stem cell research and regeneration, further establishing their significance in the scientific community [32]. Given the proposition that planarians offer promise as an alternative model for neurotoxicity [33], and recognizing the need for information regarding the toxicity of chlorpyrifos, a potent neurotoxic insecticide, particularly concerning indigenous species, our investigation focuses on its impact on *Girardia tigrina*, a planarian species indigenous to Brazilian freshwaters. Thus, the primary objective of this study is to evaluate the acute and chronic toxicity of the insecticide chlorpyrifos on freshwater planarians *G. tigrinia* using mortality, behaviour, regeneration, and reproduction as endpoints.

## 2. Materials and Methods

### 2.1. Test Organisms

A planarian *G. tigrina* culture was kept at the Plant Metabolism and Ecotoxicology Laboratory of Instituto Federal Goiano—Campus Rio Verde, in American Standard Test and Materials aqueous medium (ASTM) [34], with constant aeration under a temperature of 22 ± 1 °C, in the absence of light. Once a week, these organisms were fed, ad libitum, with bovine liver, and the medium was renewed immediately after the animals had finished feeding. A week before testing, the organisms were deprived of food to prevent digesting food from meddling with the experiment’s outcomes and guarantee consistency in toxicity responses [35].

### 2.2. Chlorpyrifos Insecticide

Chlorpyrifos was prepared from a liquid commercial formulation (Clorpirifós®; Fersol 480 EC; Manufactured by Ameribrás – Rodovia Raposo Tavares – Km 22,5, Ed. The Square – Sala 03 / Bloco B; CEP: 06709-015; Cotia/São Paulo, Brazil), with 480 g L^−1^ of active ingredient (a.i.) concentration. A stock solution of 100 g a.i. L^−1^ was prepared in distilled water. The stock solution was protected from light and stored at 4 °C to prevent degradation. Experimental solutions were prepared by serial dilution of the stock solution in ASTM medium.

### 2.3. Acute Trials

**Figure 1:**
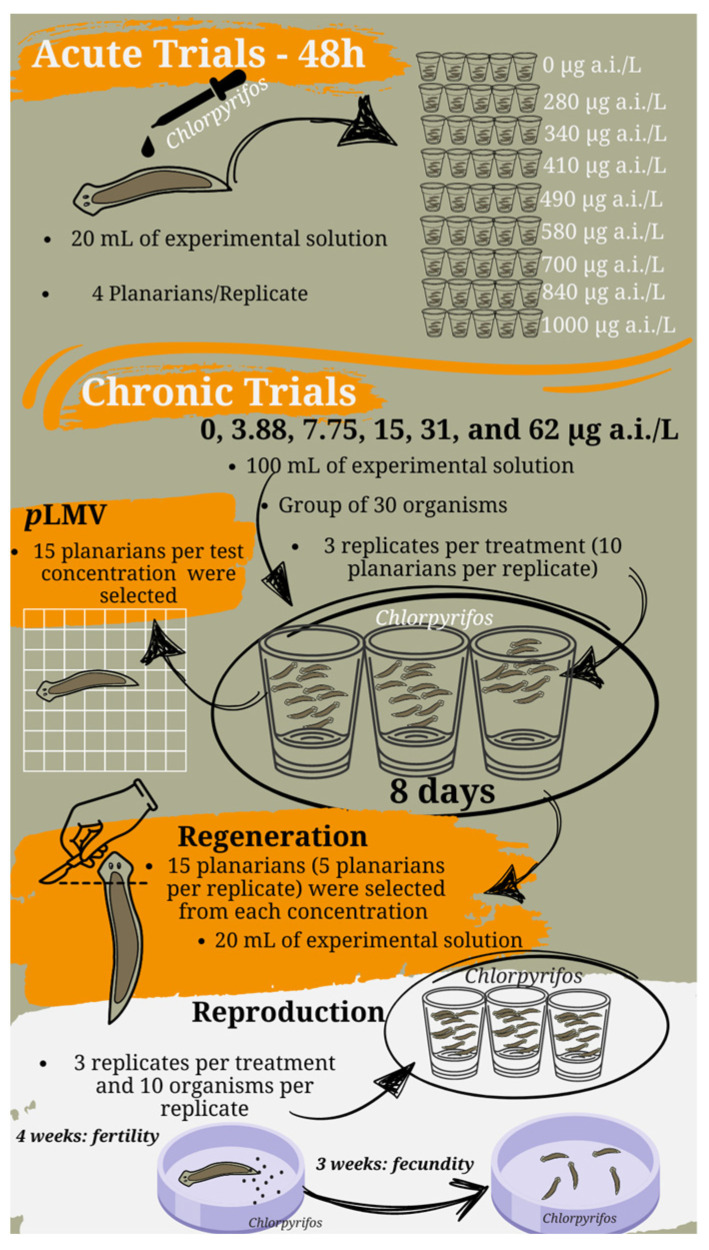
Infographic illustrating the experimental design for acute and chronic exposures.

Eight nominal concentrations (280, 340, 410, 490, 580, 700, 840, and 1000 µg a.i. L^−1^) were used for acute toxicity tests (Figure 1). The tests were carried out in polyethylene terephthalate (PET) flasks containing 20 mL of experimental solution at 22 ± 1 °C. Five replicates were prepared for each concentration, and each flask contained four planarians (1.0 ± 0.2 cm full length). All test solutions were serial dilutions prepared using ASTM medium. The lethal concentration (LC_50_) for *G. tigrina* was determined after 48 h of exposure to chlorpyrifos, and compared to the control treatment (ASTM medium).

### 2.4. Chronic Trials

To evaluate the locomotion and regeneration process, planarians of 1.0 ± 0.2 cm full length were exposed to different chlorpyrifos concentrations for eight days, with the following nominal concentrations, 3.88, 7.75, 15, 31, and 62 µg a.i. L^−1^, and a control treatment containing only ASTM medium (Figure 1). The tests were conducted under dark conditions at 22 ± 1 °C, and the planarians were deprived of food one week before and during the experimental test. The exposure was carried out with a group of 30 organisms divided into three replicates per treatment (10 planarians per replicate) in glass beakers containing 100 mL of experimental solution. The test solutions with the respective concentrations were renewed every 4 d during exposure, which lasted 8 d. After eight days of exposure to the respective concentrations, the effects on locomotion and regeneration were assessed as described below.

#### 2.4.1. Planarian Locomotor Velocity (*p*LMV)

To evaluate planarian locomotor velocity (*p*LMV), 5 planarians from each of the 3 replicates per test concentration (totalling 15 planarians per test concentration) were selected. A transparent, round container was positioned on a graph sheet with lines spaced at 0.5 cm intervals. The container bottom was filled with ASTM medium, which was sufficient to cover the planarians. Following a thirty-second adjustment period, each planarian’s movement was observed. The distance covered was recorded as the number of gridlines crossed and re-crossed over a two-minute period. The mean number of gridlines covered by planarians per replicate (from each of the 3 replicates) per minute was obtained, and after, mean distance covered per minute per concentration was calculated to obtain the *p*LMV (adapted from [28,36,37]).

#### 2.4.2. Regeneration

Fifteen planarians (5 planarians per replicate) were selected from each concentration and were decapitated with a single cut behind the auricles. After decapitation, the planarians were transferred to a PET flask with 20 mL of the respective experimental solution. The number of hours required for the formation of new photoreceptors, auricles, and the complete head was monitored using a stereomicroscope and recorded (adapted from [27,37]).

#### 2.4.3. Reproduction

For the reproduction test, the organisms were exposed to the insecticide for 4 weeks to assess fertility and 3 weeks to assess fecundity (Figure 1). The planarians used were adults (1.5 ± 0.1 cm in length). As described for the previous parameters, five nominal concentrations (3.88, 7.75, 15, 31, and 62 µg a.i. L^−1^) were used with 3 replicates per treatment and 10 organisms per replicate, followed by a control treatment with ASTM medium only. These organisms were introduced into PET flasks containing 100 mL of experimental solution. Weekly, the solutions of each concentration were renewed after feeding the organisms with bovine liver (*ad libitum*). The experiment was carried out at 22 ± 1 °C, in the absence of light, and observed daily. Fecundity per test concentration was evaluated by the number of cocoons produced per day and divided by the number of planarians exposed. Fertility per test concentration was determined by the number of offspring (planarians that hatched from the cocoons) divided by the number of cocoons produced (adapted from [38,39]).

### 2.5. Statistical Analysis

The acute toxicity of chlorpyrifos in *G. tigrina* was determined by estimating the lethal concentration causing 50% mortality (LC_50_) using a dose-response curve. To assess chronic toxicity parameters, analyses of variance (ANOVA) were conducted on the data obtained from planarians exposed to chlorpyrifos. Subsequently, Dunnett’s post hoc test was applied to assess significant differences when compared to the control treatments. To verify whether the data agreed with ANOVA assumptions, locomotion and reproduction data were analysed for homogeneity of variances and normality by using the Bartlett and Kolmogorov–Smirnov tests, respectively. The regeneration tests were not in accordance with the assumptions of ANOVA analysis. Therefore, it was necessary to use nonparametric statistics using the Kruskal–Wallis test (Dunns’ post hoc test). Statistical analyses were performed using GraphPad Prism software version 7.0 (GraphPad Software, La Jol-la, CA, USA).

## 3. Results

### 3.1. Acute Effect of Chlorpyrifos on Planarians

**Figure 2:**
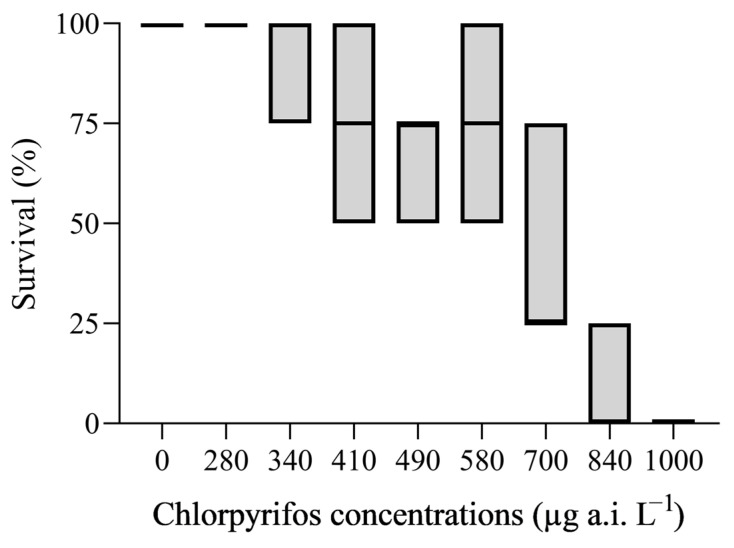
Percentage of survival of *G. tigrina* after 48 h exposure to chlorpyrifos.

The 48 h LC_50_ (95% IC) of chlorpyrifos for *G. tigrina* was 622.8 µg a.i. L^−1^ (minimum 582 and maximum 664.8 µg a.i. L^−1^). At the end of the exposure, no mortality was observed in the control treatment (Figure 2).

### 3.2. Chronic Effects of Chlorpyrifos in Planarians

**Figure 3:**
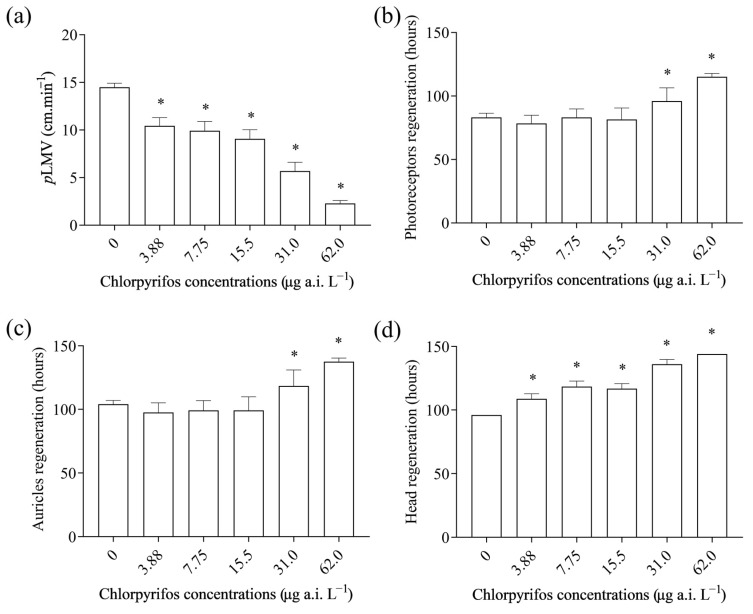
Effects of sublethal concentrations of chlorpyrifos on *G. tigrina* after eight days of exposure: (**a**) The locomotor velocity (*p*LMV); (**b**) photoreceptor regeneration; (**c**) regeneration of the auricles; (**d**) complete head regeneration. Data are presented as mean ± standard error of mean. * Significant difference is observed in comparison with the control treatment (Dunns’ post hoc test).

Locomotion velocity (*p*LMV) of *G. tigrina* decreased significantly with increasing concentrations of chlorpyrifos when compared to the control treatment (F_(5, 84)_ = 28.29; *p* < 0.0001). The lowest observed effect concentration (LOEC) for *p*LMV was 3.88 µg a.i. L^−1^ (Figure 3a).

Regarding the regeneration process, the planarians exposed to increasing concentrations of the chlorpyrifos-based insecticide exhibited significant delay in complete regeneration (F_(5, 84)_ = 28.5; *p* < 0.0001; Figure 3d), and photoreceptor (H = 37.49; *p* < 0.0001; Figure 3b) and auricle formation (F_(5, 84)_ = 3.663; *p* > 0.05; Figure 3c) when compared to the control treatment. The LOEC for complete head regeneration was determined to be 3.88 µg a.i. L^−1^. A NOEC of 15.5 µg a.i. L^−1^ and a LOEC of 31.0 µg a.i. L^−1^ were observed for both photoreceptor and auricle regeneration. 

**Figure 4:**
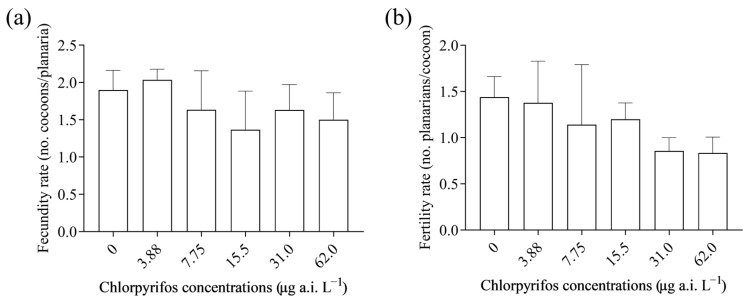
Effects of sublethal concentrations of chlorpyrifos on the reproduction of *G. tigrina*. (**a**) Fecundity rate. (**b**) Fertility rate. Data are presented as mean ± standard error of mean.

**Figure 5:**
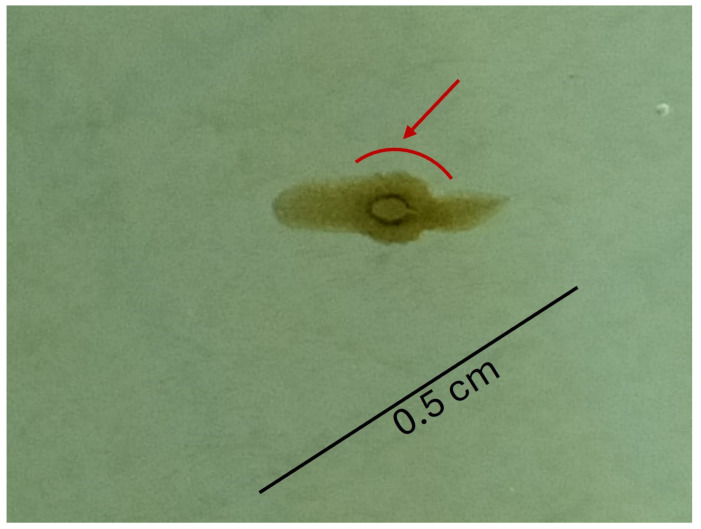
Injury observed in planarians exposed to chlorpyrifos 62 µg a.i. L^−1^ on the 15th day of the reproduction experiment. The red arrow and line highlight the area injured.

Planarian reproduction was evaluated in terms of fecundity rate and fertility rate. No significant reduction in fecundity (F_(5, 12)_ = 0.514; *p* > 0.05; Figure 4b) or in fertility (F_(5, 84)_ = 3.979; *p* > 0.05; Figure 4b) rates were observed in planarians, despite a slight decrease observed for the fertility rate at the higher tested concentrations. Lesions were observed in planarians exposed to 62 µg a.i. L^−1^ (Figure 5).

## 4. Discussion

This study provides relevant information on the toxic effects of chlorpyrifos on non-target tropical freshwater invertebrates *G. tigrina*, affecting the individual responses of the organisms (decreasing locomotor activity and delayed regeneration), in addition to survival. These effects can have potentially deleterious impacts on the planarians’ population. This knowledge about the collateral effects caused by chlorpyrifos in non-target aquatic organisms is of fundamental importance for the assessment of its impacts and risks in freshwater ecosystems. Studies for organisms from tropical regions already exist [40], but there is increasing awareness that organisms in temperate climates may have differences in susceptibility to chemicals [10]. 

Although the literature provides insights into the acute and chronic toxicity of chlorpyrifos in aquatic organisms [20] and planarians *D. dorotocephala* [41] and *D. japonica* [33,42,43,44], only one study examined its acute effects [45], but not chronic toxicity, on the planarian *G. tigrina*, which is present in Brazil. Our study showed that the insecticide chlorpyrifos was toxic to *G. tigrina* after exposure for 48 h with a LC_50_ of 622.8 µg a.i. L^−1^. Lares et al. (2023) [45] reported a LC_50_s of 2.64 and 5.55 mg L^−1^ to bigger animals (18 mm) collected in two different locations of Argentinian Patagonia. Therefore, the difference in the animals’ developmental stage and a previous history of pesticide exposure may explain the higher tolerance to chlorpyrifos reported. Even the studies with other planarians *D. japonica* showed a LC_50_ of 39 and 31.0 µM (13.7 and 10.9 µg L^−1^) chlorpyrifos (Dursban) after 12 days exposure in intact and regenerating worms, respectively [43], while *D. dorotocephala* light strain and dark strain showed a 7-day exposure LC_50_ of 2.0 mg L^−1^ and 2.2 mg L^−1^, respectively, at 27 °C [41]. The present study confirms that the planarian *G. tigrina*, like *D. japonica*, exhibited a higher sensitivity to the insecticide chlorpyrifos when compared to *D. dorotocephala* and various other freshwater invertebrate organisms. Specifically, the LC_50_ values for chlorpyrifos in *G. tigrina* were lower than those reported for rotifer *Brachionus calyciflorus* at 6665.80 µg L^−1^; molluscs *Lanistes carinatus* at 2710.00 µg L^−1^, *Bulinus truncatus* at 1320.00 µg L^−1^, and *Pomacea canaliculata* at 978.00 µg L^−1^; insects *Chironomus riparius* at 2774.00 µg L^−1^, *Sialis lutaria* at 21,700.00 µg L^−1^, and *Anopheles sinensis* at 4700.00 µg L^−1^; crustacean *Neocaridina denticulata* at 692.91 µg L^−1^, and even the fish *Anguilla anguilla* at a 48 h LC_50_ of 690.00 µg L^−1^ and the parasitic nematode *Agamermis unka*, under a static exposure in freshwater medium, with a LC_50_ of 1210.00 µg L^−1^) [22]. 

Exposure to chlorpyrifos also caused a reduction in planarian locomotor activity. *G. tigrina* behaviour was reduced after 8 days of exposure to chlopyrifos with a LOEC of 3.88 µg a.i. L^−1^. The results from our study agree with previous studies where exposure to chlorpyrifos of other species of freshwater planarian *D. japonica* and *D. dorotocephala* caused alterations in their behaviour [33,43]. Similarly, chlorpyrifos exposure decreased locomotor performance in about 50 % of *Rhinella arenarum* tadpoles at 75 µg L^−1^ [46], paralysed and reduced instinctive behavioural responses in freshwater fish *Cyprinus carpio* and the frystage of guppy *Poecilla reticulata* [23,47], and altered locomotor activity of agile frog *Rana dalmatina* tadpoles [48]. The alterations in planarian locomotor activity may be related to changes in body surface and neurotoxicity associated with exposure to chemical agents, since the planarians move by sliding, and this activity is totally muscular [49,50]. Additionally, the reduction in the locomotor activity of exposed planarians may be associated with effects of chlorpyrifos on the cholinergic system of these planarians, as chlorpyrifos’ main mechanism of toxicity is the inhibition of acetylcholinesterase (AChE) activity [51]. This inhibition can disrupt neurotransmission and neuromuscular function, directly affecting locomotion and regeneration in planarians. The inhibition of AChE activity in planarian *D. japonica* was reported and associated with reducing negative thermotaxis behaviour [42]. The reduction in the locomotion of planarians can also imply the inability of planarians to move away from unfavourable environments/conditions and a greater vulnerability to attack by predators [36,52].

The results also showed a delay in the regeneration of exposed planarians. Planarian regeneration involves processes such as stem cell proliferation and cell differentiation [30], and chlorpyrifos could possibly induce neurotoxic developmental effects like other organophosphates [31] by interfering with the regeneration process. The delayed regeneration of the planarians’ head may have negative effects on planarians at the organismal level as well as at the population level. Indeed, planarians detect the presence of prey nearby using their auricles, which have chemotactic roles [53]. On the other hand, planarians’ photoreceptors are used for the perception of light, and these organisms are negatively phototropic [54]. Thus, any delay in the regeneration of auricles can affect their ability to locate prey and feed, while a delay in the regeneration of photoreceptors can disrupt their ability to accurately perceive light intensity and negative phototactic responses. Furthermore, chlorpyrifos, through its developmental neurotoxic effects, might have altered neural activities during the regeneration of photoreceptors, which resulted in the delay observed [31].

The observed effects of chlorpyrifos on *G. tigrinia*, including the delay in locomotion and regeneration, suggest a potential impact on the nervous system of these planarians. Neurotoxicity in the form of alterations in brain morphology due to chlorpyrifos exposure has been reported in *D. japonica* [33]. Chlorpyrifos is known to target the nervous system of insects, leading to neurological disorders and coordination problems [51]. This mode of action could explain the observed negative effects on locomotion in planarians. Therefore, our findings reinforce the possible role of chlorpyrifos in influencing complex regeneration processes akin to embryonic development, through effects on cell replication and neurotropic signalling pathways. These effects likely contribute to the observed delay in planarian regeneration, hypothesizing that chlorpyrifos interferes with fundamental cellular differentiation processes necessary for the reconstruction of lost structures.

Moreover, the results of this study suggest a reduction in the planarian fertility rate, though the effects were not significant. In addition, there was no effect on the fecundity rate of the exposed planarians at the tested concentrations of chlorpyrifos. Similarly, guppy (*Poecilia reticulata*) exposure to chlorpyrifos was implicated in a reduction in the number of offspring and offspring survival of guppy [47]. Effects of chlorpyrifos on the fertility rate of planarians might impact the natural population of planarians in the freshwater ecosystem.

Future studies focusing on the nervous system of *G. tigrina* exposed to the pesticide chlorpyrifos are of fundamental importance to further understand the specific mechanisms of toxicity. While some studies have explored chlorpyrifos’ lethal and sublethal toxicity in other organisms, assessing its effects at the physiological, morphological, biochemical, and survival levels and the specific evaluation of planarians’ responses to chlorpyrifos remain limited; thus, studies on chlorpyrifos effects on these will be considered in the future.

## 5. Conclusions

In conclusion, this study provides valuable insights into the chronic toxicity of chlorpyrifos in *G. tigrina*, specifically regarding its behavioural and regenerative effects. The observed effects occurred at concentrations below or comparable to the predicted levels of chlorpyrifos in aquatic environments, such as 37.3 µg a.i. L^−1^. These data reinforce the importance of the biomonitoring of this insecticide in natural aquatic systems and demonstrate the potential use of planarians as a sensitive species for assessing environmental contamination by chlorpyrifos. Moreover, the chronic effect observed at environmentally relevant concentrations underscores the importance of this study for contributing to the ecological risk assessment of chlorpyrifos in freshwater ecosystems. Ultimately, these findings contribute to the broader understanding of the environmental risks associated with chlorpyrifos and support informed decision-making and management strategies aimed at protecting freshwater ecosystems.

## Data Availability

All data generated or analysed during this study are included in this published article.
